# Genome-wide identification of *ZF-HD* gene family in *Triticum aestivum*: Molecular evolution mechanism and function analysis

**DOI:** 10.1371/journal.pone.0256579

**Published:** 2021-09-24

**Authors:** Hongli Niu, Pengliang Xia, Yifeng Hu, Chuang Zhan, Yiting Li, Shuangjun Gong, Yan Li, Dongfang Ma

**Affiliations:** 1 Hubei Collaborative Innovation Center for Grain Industry/Engineering Research Center of Ecology and Agricultural Use of Wetland, Ministry of Education/College of Agriculture, Yangtze University, Jingzhou, China; 2 Enshi Tobacco Company of Hubei Province, Enshi, China; 3 Key Laboratory of Integrated Pest Management on Crop in Central China, Ministry of Agriculture/Hubei Province Key Laboratory for Control of Crop Diseases, Pest and Weeds/Institute of Plant Protection and Soil Science, Hubei Academy of Agricultural Sciences, Wuhan, China; ICAR-Indian Institute of Wheat and Barley Research, INDIA

## Abstract

*ZF-HD* family genes play important roles in plant growth and development. Studies about the whole genome analysis of *ZF-HD* gene family have been reported in some plant species. In this study, the whole genome identification and expression profile of the *ZF-HD* gene family were analyzed for the first time in wheat. A total of 37 *TaZF-HD* genes were identified and divided into TaMIF and TaZHD subfamilies according to the conserved domain. The phylogeny tree of the TaZF-HD proteins was further divided into six groups based on the phylogenetic relationship. The 37 *TaZF-HDs* were distributed on 18 of 21 chromosomes, and almost all the genes had no introns. Gene duplication and Ka/Ks analysis showed that the gene family may have experienced powerful purification selection pressure during wheat evolution. The qRT-PCR analysis showed that *TaZF-HD* genes had significant expression patterns in different biotic stress and abiotic stress. Through subcellular localization experiments, we found that TaZHD6-3B was located in the nucleus, while TaMIF4-5D was located in the cell membrane and nucleus. Our research contributes to a comprehensive understanding of the *TaZF-HD* family, provides a new perspective for further research on the biological functions of *TaZF-HD* genes in wheat.

## Introduction

The developmental processes of plant are controlled by various regulatory proteins. The transcription factors (TFs) are one kind of special regulatory proteins which regulate other gene expression, and they are characterized by one or more DNA binding domains (DBDs) [1]. TFs can bind with specific DNA sequences to regulate the response of plants to environmental stimuli, and further play important roles in plant growth and development processes [2]. The Zinc finger homeodomain (ZF-HD) TFs, containing a conserved zinc finger (ZF) domain in N-terminal and a homeodomain (HD) in C-terminal, are members of plant-specific TF super family [3].

The homeodomain (HD) of ZF-HDs is a highly conserved domain in the homeobox gene family, with approximately 60 conserved amino acids [4, 5]. The homeodomain fold into a characteristic three-helix structure called recognition helix, which attaches to the main sulcus of DNA to form a special connection with DNA [6]. HD proteins are widely found in animals and plants, and participate in protein-protein interactions or other regulatory functions [7]. Proteins with homeodomains can be divided into six different families: leucine zipper-associated HD (HD-Zip), zinc finger motif-associated HD (ZF-HD), WUSCHEL-related homeobox (WOX), Bell-type HD, finger-like domain associated with an HD (PHD finger), and knotted-related homeobox (KNOX) proteins [8].

The zinc finger, as an important motif, is widely present in various regulatory proteins [9], and play an important role in the formation of homo-dimerization or hetero-dimerization between members of the ZF-HD family [10]. A typical zinc finger motif contains two pairs of conserved cysteine residues and/or histidine residues, which coordinates with a single zinc ion to form the finger-like loop [11]. Most zinc finger structures participate in DNA binding and protein interactions, and a few of them participate in RNA binding and protein folding [12, 13]. Depending on the nature, number, and spatial structure of the residues that bind to zinc, zinc finger structures can be divided into different categories, such as C3H, C2H2, PHD, C2C2, LIM and RING-finger [14–16]. Previous studies have shown that the ZF-HD family could be divided into two subfamilies, Zinc finger-homeodomain (ZHD) and MINI ZINC FINGER (MIF) [4]. Three MIF genes were firstly identified in *Arabidopsis*, and their encoding proteins were similar to the ZF domain of the ZF-HD proteins but lacks the HD domain [17].

So far, growing studies in bioinformatics and expression pattern analysis of the ZF-HD transcription factor family in other plants have been reported, such as *Arabidopsis* [4], tartary buckwheat [18], Chinese cabbage [3], grapes [19], upland cotton [20] and maize [21]. A new class of zinc finger homology domain proteins was firstly identified in *Flaveria trinervia*, which can indirectly regulate C4 phosphoenolpyruvate carboxylase encoding gene [10]. Moreover, ZF-HD genes also participate in responding to adverse stresses [22]. In *Arabidopsis*, overexpression of NAC and AtZHD1 could improve the drought tolerance of the plant [23]. Another research showed that AtZHD1 could specifically bind to the promoter sequence of *EARLY RESPONSE TO DEHYDRATION STRESS 1* (*ERD1*) to cope with salinity, drought, and abscisic acid treatments [23]. By inducing the overexpression of *MIF1* gene in *Arabidopsis*, researchers found that *MIF1* may participate in plant growth and development by regulating multiple hormones [17]. In addition, studies showed that most *ZF-HD* genes had a significant impact on various flowering-related exogenous hormones (GA, 6-BA), and were mainly expressed in flower tissues, indicating that they may play regulatory roles during flowering induction [24, 25]. In soybean, transient expression of *GmZF-HD1* and *GmZF-HD2* in *Arabidopsis* protoplasts confirmed that they can respond to pathogens attack and activate the gene expression of *GmCaM4* [26], which is involved in plant defense responses. In recent years, more and more studies on the ZF-HD transcription factor family have been reported. And various functions of ZF-HD transcription factor have gradually been uncovered.

Wheat is one of the most important food crops in the world, accounting for more than half of total human consumption [27, 28]. However, its safe production is seriously threatened by abiotic and biotic stresses. Therefore, improving resistance and yield is one of the most important goals of wheat breeding programs. Although *ZF-HD* has been studied in a variety of plants, systematic studies on the *ZF-HD* family of wheat have not been reported. In present study, a comprehensive identification of the *ZF-HD* genes of wheat and its three close relatives (*Triticum urartu*, *Triticum dicoccoides*, and *Aegilops Tauschii*) was conducted. Furthermore, the gene structure, subcellular localization, phylogenetic relationship, expression profiles of *ZF-HD* family genes in wheat were systematically analyzed. At the same time, the transcriptional autoactivation activity of two genes were verified by yeast two-hybrid system. In conclusion, this study lays the foundation for analyzing the function of TaZF-HD proteins, and also provides a theoretical reference for mining stress resistance genes in wheat breeding for disease resistance.

## Materials and methods

### Identification of ZF-HD family genes in wheat

A comprehensive bioinformatic method was conducted to identify the members of the ZF-HD gene family from the wheat reference genome IWGSC RefSeq v1.1 (https://wheat-urgi.versailles.inra.fr/Seq-Repository/Assemblies). The known ZF-HD protein sequences, including 17 ZF-HDs from *Arabidopsis* (https://www.arabidopsis.org) [4], 24 ZF-HDs from *Zea mays* (ZmLTP, maizeGDB, https://maizegdb.org) [21], 15 ZF-HDs from *Oryza sativa* (http://rice.plantbiology.msu.edu/index.shtml) [4], were downloaded from database. Then, the total 56 obtained protein sequences were used as query sequences for BLASTp analysis among the wheat database IWGSCv1.1 to obtain the candidate TaZF-HD protein sequences. The cutoff values (e-value < 10^−5^) were used to ensure the reliability. The repeated and unmatched candidate sequences were removed. Furthermore, these remaining candidate genes of wheat were further validated by Pfam (http://pfam.xfam.org/) (PF04770) and InterPro (https://www.ebi.ac.uk/interpro/) (IPR006456) online software. Proteins that do not exhibit ZF domain were excluded, and the members of ZF-HD gene family were finally determined.

### Construction of the phylogenetic tree and prediction of TaZF-HD protein characteristics

The amino acid sequences of 17 AtZF-HDs, 24 ZmZF-HDs, 15 OsZF-HDs, and 37 determined TaZF-HDs were aligned using ClustalW2 software [29]. Then the phylogenetic tree was constructed by the Neighbor-joining (NJ) method, and the tree was generated and decorated by Interactive Tree of Life online tool (iTOL, https://itol.embl.de/itol.cgi). The ZF-HD proteins common features, including protein length, isoelectric points (pI), molecular weights (MW), instability index, grand average of hydropathicity (GRAVY), and amino acid composition were analyzed by ExPASy Server10 (https://web.expasy.org/protparam/) [30]. Moreover, the subcellular localization of TaZF-HD proteins was predicted by Plant-mPLoc (http://www.csbio.sjtu.edu.cn/bioinf/plant-multi/) [31].

### TaZF-HDs motifs, gene structures and three-dimensional structure analysis

The conserved motifs of TaZF-HD proteins were identified using MEME Suite (http://meme-suite.org) [32] in discriminative mode. The parameters were set as: Each sequence may contain any number of non-overlapping motif; The number of different motifs up to 20, and the motif widths range from 6 to 50 amino acids. Then these motif patterns were drawn by TBtools software [33]. According to the annotation information, the gene structures of TaZF-HDs were displayed using the online software Gene Structure Display Server (GSDS2.0, http://gsds.cbi.pku.edu.cn/) [34]. The three-dimensional structures of the TaZF-HD proteins were analyzed by SWISS-MODEL (https://swissmodel.expasy.org).

### Promoter analysis of TaZF-HD genes

The 1500 bp upstream sequences of the *TaZF-HD* gene were extracted from the wheat genome. Then *cis*-elements were identified using PlantCARE online tool (http://bioinformatics.psb.ugent.be/webtools/plantcare/html/) [35]. And the results were shown using R package ‘pheatmap’.

### Chromosomal location and gene duplication analysis of TaZF-HDs

The chromosomal physical location of the *ZF-HD* genes was displayed using MapInspect software basing on the wheat reference genome annotation information. Gene duplication events were divided into tandem duplication events and segmental duplication events [36]. Tandem duplication events were identified using the following evaluation criteria: (1) Length of the aligned sequence > 80% of the length of each sequence; (2) Identity > 80%; (3) Threshold ≤ 10^−10^; (4) Only one duplication can be recognized when genes are linked closely; (5) Intergenic distance is less than 25 kb. If genes satisfied criteria (1), (2), and (3), and were located on different chromosomes, they were judged to be segmental duplications [37]. In addition, the Dna Sequence Polymorphism Version 6 (DnaSP6) was used to calculate the non-synonymous (Ka) and synonymous (Ks) substitution rates of duplicated gene pairs [38].

### Homology and synteny analysis of TaZF-HD genes

Orthologous homology genes between allohexaploid common wheat and sub-genome donor species (*T*. *urartu*, *T*. *dicoccoides*, and *Ae*. *Tauschii*) were identified by BLASTp analysis, and the cutoff values (e-value < 10^−10^, identity > 80%) were used to ensure the reliability of orthologs. The homology relationships were displayed using the R package ‘circlize’ [39]. In addition, basing on the multiple alignments among wheat and other species (*Arabidopsis*, *S*. *lycopersicum*, *Z*. *mays*, and *O*. *sativa*), the synteny relationship within species of *ZF-HDs* was analyzed using MCScanX [40].

### MicroRNA targeting and protein interaction analysis of TaZF-HD genes

MicroRNA (miRNA) has an important function of regulating gene expression, in order to identify miRNAs that target the TaZF-HD genes, we submitted the TaZF-HD gene sequences to psRNATarget (http://plantgrnbb.noble.org/psRNATarget) with default parameters [41]. And Cytoscape software (https://www.omicshare.com/tools/) was used to drew the linkage between the predicted miRNA and corresponding target genes [42]. Protein-protein interaction (PPI) can regulate a variety of biological activities and functions [43]. In order to better understand the biological functions and regulatory network of TaZF-HD genes, the online STRING database(https://string-db.org) was used to predict the PPI network.

### Multiple conditional transcriptome analysis of TaZF-HDs

Original RNA-seq data of wheat from multiple conditional transcriptome analyses were download from the NCBI Short Read Archive (SRA) database and mapped to the wheat reference genome through hisat2 [44]. After that, the expression levels of *TaZF-HDs* (FPKM, normalized fragments per kilobase of the exon model per million mapped reads) were calculated by Cufflinks [45]. The heatmap of wheat *ZF-HD* genes was generated using the R package ‘pheatmap’.

### Preparation of wheat materials and stress treatments

Seeds of Yangmai20 (a hexaploid common wheat variety) were surface sterilized with 1% hydrogen peroxide, then rinsed with distilled water, and germinated for 2 days in an incubator at 26°C [46]. The seedlings were transferred and cultured in a continuously ventilated 1/4 Hoagland nutrient solution [47]. The stress treatments in present study included three abiotic stresses (hormonal, drought, and high salt) and one biotic stress (*Fusarium graminearum*). After 5 days, the stems of wheat seedlings were inoculated with hypha of *F*. *graminearum* (PH-1). The stems were collected at 24, 48, 72, and 120 h after inoculation. Moreover, wheat seedlings were treated with 150 mM sodium chloride (NaCl), 20% polyethylene glycol (PEG), 100 μM abscisic acid (ABA). After treatments, leaves and roots were collected at 2, 4, 8, 12, 24, 72, 120 h. During the treatment, the growth environment of wheat seedlings was set at 25°C and 16 h /8 h (day/night). Three biological replicates were set up for each treatment. The samples were immediately frozen with liquid nitrogen and stored at -80°C.

### Real-time quantitative PCR and data analysis

In order to clarify the development and tissue-specific expression profiles of *TaZF-HD*, 4 genes with high expression in transcriptome data were selected to perform quantitative real-time PCR (qRT-PCR) to detect their expression levels. Gene-specific primers were designed using Primer Premier 5.0 and listed in S7 Table (Synthesized by Sangon Biotech, Shanghai, China). Total RNA in different wheat samples was extracted using TRizol (Vazyme, Nanjing, China). The cDNA was reverse transcript using the reverse transcriptase cDNA kit (Vazyme, Nanjing, China), and was diluted to 200 ng/μL with ddH_2_O. The qRT-PCR reaction system contained 10 μL of 2×SYBR green Mix, 0.4 μL of each primer (10 μM), 1 μL of template (200 ng/μL) and 8.2 μL of ddH_2_O to reach a total volume of 20 μL. The PCR amplification procedure was performed as follows: pre-denaturation at 95°C for 30 s (step 1), denaturation at 95°C for 10 s (step 2), primer annealing/extension and collection of fluorescence signal at 58°C for 30 s (step 3). From step 2 to step 3 cycles 40 times. Three technical replicates were performed for each sample. Relative expression levels were determined using the 2^–ΔΔCt^ method [48]. The ADP-ribosylation factor Ta2291, which stable expressed under various conditions was used as an internal reference gene for qRT-PCR analysis (Forward: GCTCTCCAACAACATTGCCAAC, Reverse: GCTTCTGCCTGTCACATACGC).

### Transient expression and subcellular localization analysis

In this study, *Agrobacterium*-mediated tobacco transient transformation system was used for subcellular localization analysis. The full-length sequences of *TaTaMIF4-5D* and *TaZHD6-3B* genes without stop codon were cloned, and then the genes were combined with pART27-GFP vector to construct transient expression vector pART27-TaMIF4-5D-GFP and pART27-TaZHD6-3B-GFP. Then the *Agrobacterium* (GV3101) containing recombinant vector was injected into *Nicotiana benthamiana* leaves. The fluorescence signal was observed 72 hours later using a fluorescence confocal microscope DMi8 (Leica, Germany).

### Verification of transcriptional autoactivation activity

In order to further verify whether *TaMIF4-5D* and *TaZHD6-3B* have transcriptional autoactivation activity, yeast two-hybrid (Y2H) system was used. Primers were designed for the *TaMIF4-5D* and *TaZHD6-3B* genes and the full-length sequences were cloned into the pGBKT7 vector. Then the recombinant vectors (pGBKT7-TaMIF4-5D and pGBKT7-TaZHD6-3B) and pGADT7 were transformed into yeast AH109 chemically competent cell. The vector pGBKT7-53 and pGBKT7-Lam were used as positive control and negative control, respectively. The transformed yeast cells were cultured at 30°C for three days.

## Results

### Identification of ZF-HDs and conservative domain analysis in wheat

After detecting domains by Pfam and InterProScan, we finally identified 37 *TaZF-HDs* from the wheat genome, which is relatively more in numbers than that of rice and maize. Multiple sequence alignment of the 37 proteins was performed using DNAMAN software, and the align results of conserved domains were combined the online serve WebLogo3 (http://weblogo.threeplusone.com/) (S1 Fig). Based on the domains of ZF-HD, the 37 *TaZF-HD* genes could be divided into *TaZHD* and *TaMIF* subfamily. The ZHD subfamily has ZF domain and HD domain, while MIF subfamily only has ZF domain. According to the distribution of genes on the wheat chromosome, genes of *TaZHD* and *TaMIF* subfamily were renamed (*TaZHD1-1A* to *TaZHD14-U*, *TaMIF1-3A* to *TaMIF5-5D*), and the wheat triplet genes were distinguished by chromosomal location (*TaZHD2-1A*, *TaZHD2-1B*, *TaZHD2-1D*). The results of multiple sequence alignment indicated that both HD and ZF domain are highly conserved in wheat. From the preliminary analysis of the *ZF-HD* gene domain by Pfam and DNAMAN, the *ZF-HD* family members obtained in this study were highly reliable and could be used for subsequent bioinformatics analysis.

### Construction of the phylogenetic tree and prediction of TaZF-HD protein characteristics

**Fig 1:**
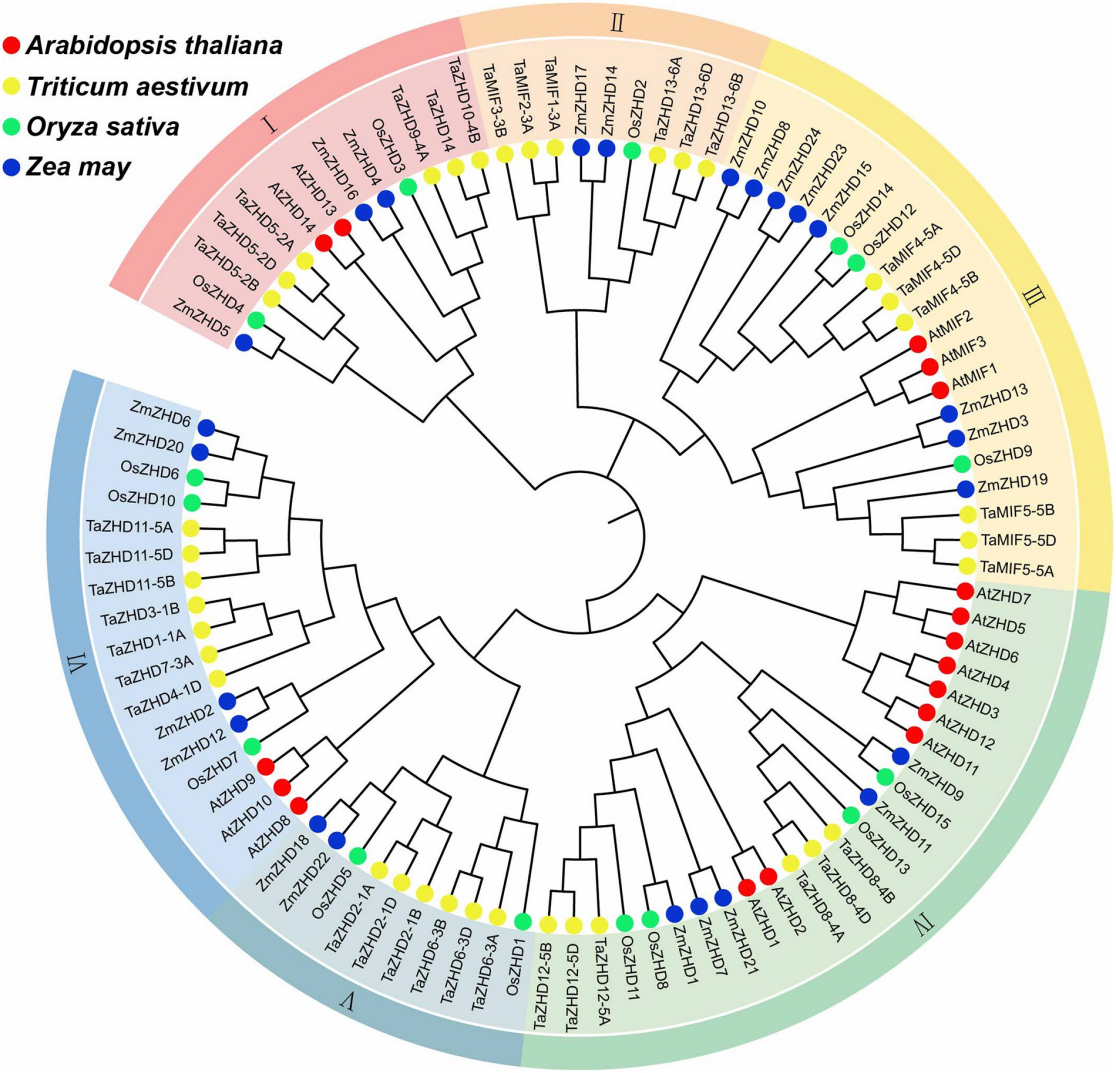
Phylogenetic tree of ZF-HDs predicted in wheat and those previously identified in maize, rice, and *Arabidopsis*. All sequences were aligned using ClustalW2, and the phylogenetic tree was constructed using the Neighbor-joining (NJ) method with 1000 bootstrap replications. Different group was distinguished by different color. ZF-HDs from different species are distinguished with different color dots.

To better understand the evolutionary history and relationships of the ZF-HD genes in wheat, a phylogenetic tree was generated using NJ method with the full-length amino acid sequences from *Arabidopsis*, rice, maize, and wheat. According to the phylogenetic tree (Fig 1), the *TaZF-HD* family could be clustered into six groups, named as Groups I-VI. Group IV contained the most members of ZF-HD proteins (24), followed by group III with 20 members. Moreover, Group V included the least members (10). Group I, IV, V, and VI only contained ZHD subfamily members, group II had three members of TaZHD subfamily and TaMIF subfamily, respectively, and group III had only TaMIF subfamily members.

The prediction results of 37 protein features and subcellular localization were listed in Table 1. The length of protein sequences of the TaZF-HDs was between 93–465 amino acids. Among them, because the members of the MIF subfamily contained only one ZF domain, their protein sequences are shorter than ZHD subfamily, and the longest sequence in MIF subfamily was 96 amino acids. The predicted molecular weight (MW) of ZF-HDs was ranging from 9.84197 kDa to 50.6004 kDa. The theoretical isoelectric point (pI) of the ZF-HD proteins was range from 6.17 to 9.89. In this study, most ZF-HD proteins were alkaline proteins or weak alkaline proteins. At the same time, only one of 37 genes had an instability index lower than 40, which was relatively stable. The GRAVY value was range from -1.14 to -0.309, which showed that all members of the ZF-HD family were hydrophilic proteins. According to the results of subcellular localization, all the predicted genes were located in the nucleus except *TaZF-HD4*-*1D*, which was located in chloroplast.

**Table 1:** Characterization of ZF-HD proteins in wheat.

Name	Accession numbers	Amino acid	Molecular weight (KDa)	Theoretical pI	Instability index		Grand average of hydropathicity (GRAVY)	Predicted location(s)
TaZHD1-1A	TraesCS1A02G087700.1	286	32.08414	8.62	73.4	unstable	-0.873	N
TaZHD2-1A	TraesCS1A02G418600.1	225	24.92645	9.41	75.23	unstable	-1.14	N
TaZHD2-1B	TraesCS1B02G448600.1	252	27.59062	9.64	71.92	unstable	-0.978	N
TaZHD2-1D	TraesCS1D02G426300.1	250	27.37345	9.89	71.81	unstable	-0.981	N
TaZHD3-1B	TraesCS1B02G106800.1	290	32.18401	7.67	78.65	unstable	-0.888	N
TaZHD4-1D	TraesCS1D02G064300.1	173	19.85822	9.83	49.15	unstable	-0.487	C
TaZHD5-2A	TraesCS2A02G305400.1	295	30.4802	6.96	65.08	unstable	-0.486	N
TaZHD5-2B	TraesCS2B02G322200.1	293	30.32005	7.26	63.66	unstable	-0.483	N
TaZHD5-2D	TraesCS2D02G303900.1	293	30.31003	7.26	64.9	unstable	-0.493	N
TaZHD6-3A	TraesCS3A02G226100.1	235	25.89576	9.13	94.81	unstable	-0.978	N
TaZHD6-3B	TraesCS3B02G258400.1	232	25.46825	8.54	92.84	unstable	-0.961	N
TaZHD6-3D	TraesCS3D02G224100.1	236	25.88365	8.22	95.47	unstable	-0.97	N
TaZHD7-3A	TraesCS3A02G308700.1	185	20.89259	8.16	73.22	unstable	-0.952	N
TaZHD8-4A	TraesCS4A02G226800.1	403	42.3537	8.64	68.72	unstable	-0.561	N
TaZHD8-4B	TraesCS4B02G089700.1	397	42.00036	8.17	73.66	unstable	-0.558	N
TaZHD8-4D	TraesCS4D02G086300.1	389	41.03615	8.17	71.49	unstable	-0.57	N
TaZHD9-4A	TraesCS4A02G264100.1	236	24.70953	8.45	70.65	unstable	-0.703	N
TaZHD10-4B	TraesCS4B02G050800.1	236	24.6795	8.45	70.65	unstable	-0.692	N
TaZHD11-5A	TraesCS5A02G227100.1	383	40.21233	8.87	73.26	unstable	-0.56	N
TaZHD11-5B	TraesCS5B02G225700.1	390	40.88114	8.5	76.78	unstable	-0.54	N
TaZHD11-5D	TraesCS5D02G234600.1	387	40.51166	8.71	71.85	unstable	-0.517	N
TaZHD12-5A	TraesCS5A02G246500.1	297	31.00971	7.29	58.5	unstable	-0.527	N
TaZHD12-5B	TraesCS5B02G243800.1	297	31.01366	7.01	56.03	unstable	-0.531	N
TaZHD12-5D	TraesCS5D02G253100.1	296	30.96664	7.01	55.62	unstable	-0.534	N
TaZHD13-6A	TraesCS6A02G273700.1	465	50.51136	6.67	97.13	unstable	-0.954	N
TaZHD13-6B	TraesCS6B02G301000.1	461	50.37821	6.64	96.06	unstable	-0.979	N
TaZHD13-6D	TraesCS6D02G253700.1	465	50.6004	6.64	95.5	unstable	-0.962	N
TaZHD14-U	TraesCSU02G070700.1	236	24.69747	8.15	70.65	unstable	-0.7	N
TaMIF1-3A	TraesCS3A02G448000.1	96	10.63383	6.17	42.14	unstable	-0.609	N
TaMIF2-3A	TraesCS3A02G448100.1	96	10.63383	6.17	42.14	unstable	-0.609	N
TaMIF3-3B	TraesCS3B02G484100.1	96	10.56172	6.49	37.2	stable	-0.624	N
TaMIF4-5A	TraesCS5A02G142700.1	94	9.856	6.88	60.27	unstable	-0.478	N
TaMIF4-5B	TraesCS5B02G141400.1	94	9.84197	6.87	54	unstable	-0.478	N
TaMIF4-5D	TraesCS5D02G149400.1	94	9.84197	6.87	54	unstable	-0.478	N
TaMIF5-5A	TraesCS5A02G227200.1	93	10.23144	7.55	63.16	unstable	-0.338	N
TaMIF5-5B	TraesCS5B02G226000.1	93	10.14835	6.93	56.89	unstable	-0.309	N
TaMIF5-5D	TraesCS5D02G234700.1	93	10.23144	7.55	63.16	unstable	-0.338	N

Note: N = Nuclear; C = Chloroplast.

### Conserved motifs, gene structure and three-dimensional structure analysis

**Fig 2:**
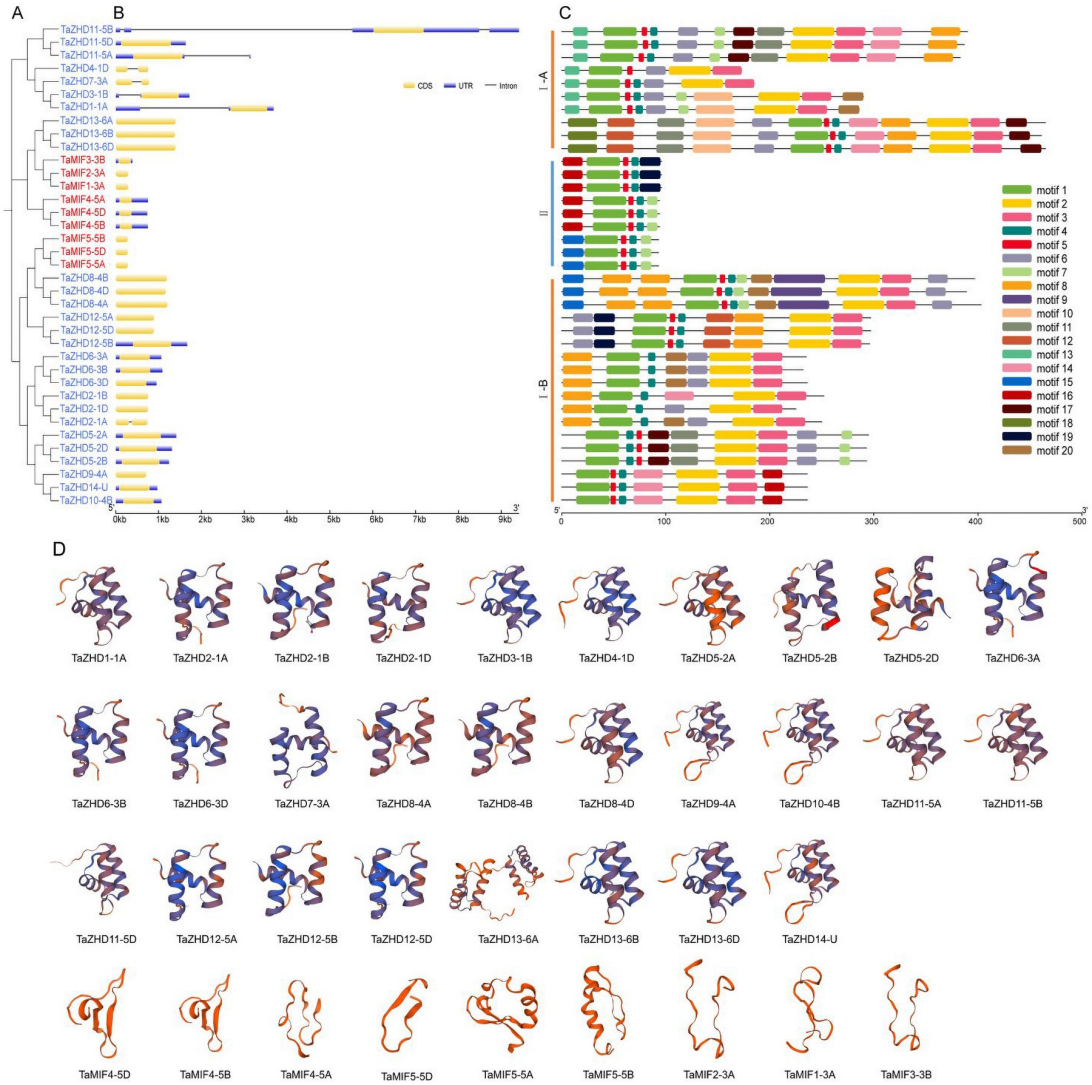
Phylogenetic tree, gene structure and motif analysis of wheat ZF-HD family. (**A**) The phylogenetic tree of TaZF-HDs. The tree was created using the Neighbor-joining (NJ) method. (**B**) The exon-intron structure of the *TaZF-HDs*. Exon-intron structure analyses were conducted using the GSDS server. The untranslated regions (UTRs) are indicated by blue boxes, the exons are indicated by yellow boxes and the introns are indicated by black lines. (**C**) The motifs of TaZF-HDs were identified by MEME tool. Each motif is indicated with a specific color. (**D**) Tertiary structure prediction of TaZF-HD proteins.

Motif is a highly conserved amino acid sequence. It is generally believed that a conserved motif will make a protein have a corresponding function or structure. According to the predicted motif of the MEME server (Fig 2C), it can be seen that although the protein length of the TaZF-HD genes ranged from 93 to 465 amino acids, the members of the TaZF-HD family had similar conserved motifs. For example, all members of ZF-HD family had motif 1, which showed that motif 1 may represent the ZF domain. And all the ZHD subfamily members had motif 2 and motif 3, which represent the HD domain.

In order to study the intron-exon structure of the *TaZF-HD* genes, the gene structures were drawn using GSDS2.0 based on the *TaZF-HD* genome annotation information. The result showed that many *TaZF-HD* genes lack introns, only seven *TaZF-HD* genes have introns, while all members of the TaMIF subfamily had no introns (Fig 2B). Among the 37 *TaZF-HDs*, *TaZHD11-5B* had the longest intron sequence and the longest untranslated regions (UTR), 16 of the 37 genes only had coding sequence (CDS). The lengths of the CDS in the same subfamily were highly similar, indicating that the *TaZF-HDs* structures were relatively conserved.

The SWISS-MODEL in the intensive mode was used to analyze the three-dimensional structure of the 37 TaZF-HD proteins. The result showed that the three-dimensional structures of the same subfamily are very similar. And the structures of the ZHD subfamily members are more complex than that of the MIF subfamily members, which is because the proteins of the ZHD family have longer sequences (Fig 2D).

### Key cis-elements in the promoters of TaZF-HD genes

**Fig 3:**
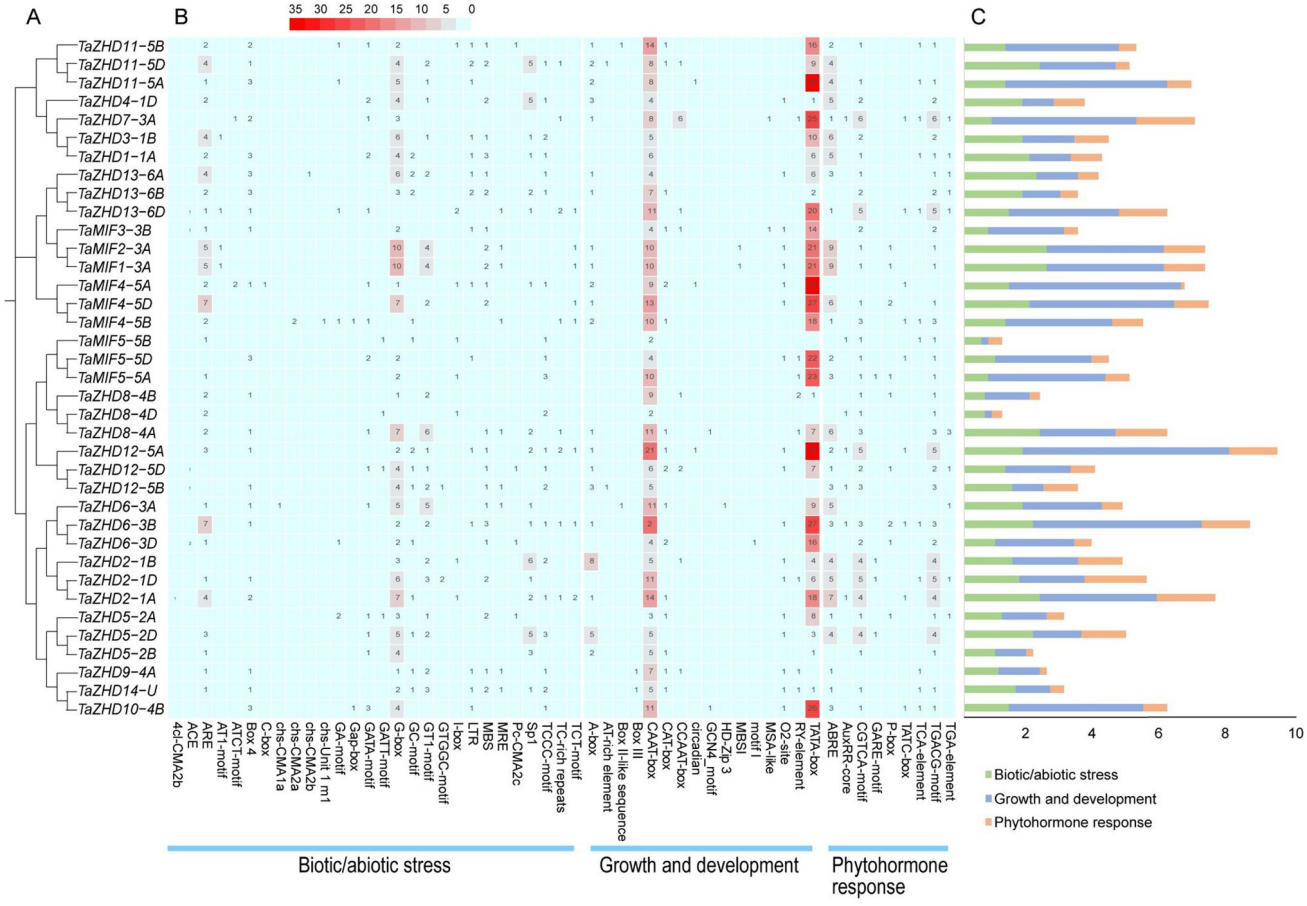
Analysis of *cis*-acting elements in the promoter sequences of *TaZF-HD* genes. (**A**) Phylogenetic analysis of wheat *ZF-HD* family; (**B**) *Cis-*acting elements in the promoters of *TaZF-HD* genes. The different colors and numbers of grids indicate the numbers of different promoter elements. (**C**) The histograms of different colors represent the sum of the *cis-*acting elements in each category.

The *cis*-acting regulatory element is a specific motif that can be bound with appropriate transcription factors to regulate gene transcription in plants, and facilitate plant response to environmental stimuli [49]. We could identify the differences in function and regulation basing on the distribution of different *cis*-acting elements in promoter region of genes. To identify putative *cis*-acting elements in the promoter region of *TaZF-HDs*, the 1500 bp upstream regions of genes before transcriptional start site (ATG) were scanned. The promoters of *TaZF-HDs* contained 53 kinds of putative response factors, including G-box involved in light responsiveness, CAAT and TATA-box involved in growth and development, ABRE involved in the abscisic acid responsiveness, TGA-element related to auxin-responsive, GARE-motif and P-box involved in gibberellin-responsive, etc (Fig 3). Among of these *cis*-acting elements, the types of *cis*-elements related to biotic/abiotic stress were the most, but the most abundant *cis*-elements were related to growth and development. Furthermore, the most widely distributed element was CAAT-box, which was contained in all TaZF-HD genes. Also, the TATA box, though it is not present in all genes, it has the largest total number. In the members of the *TaZF-HD* family, the promoter upstream sequence of *TaZHD12-5A* had the largest number of responsive elements (91); while *TaMIF5-5B* and *TaZHD8-4D* only contained 11 elements each.

### Chromosomal location and gene duplication analysis of *TaZF-HDs*

**Fig 4:**
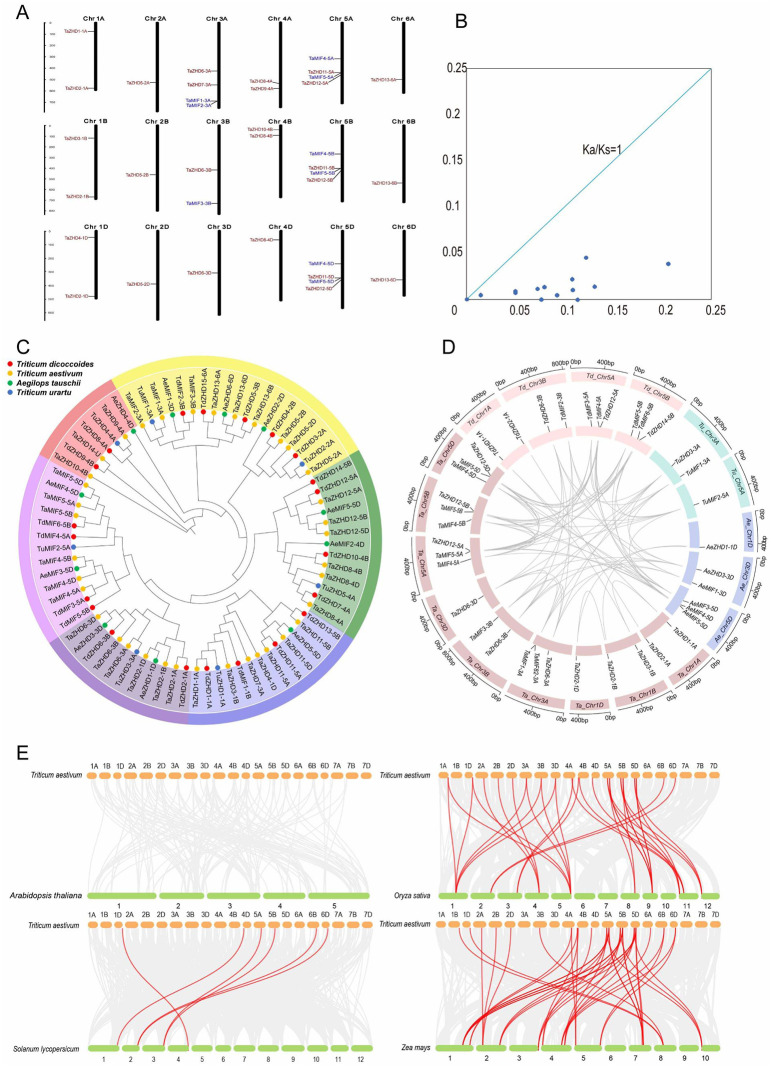
Chromosomal locations, Ka/Ks analysis of *TaZF-HDs* and orthologous analysis between wheat and other species. (**A**) Chromosomal locations of *TaZF-HDs*. The scale on the left indicates the length of the wheat chromosome, different colors of the genes represent different subfamily, red letters represent the genes of the ZHD subfamily, and the blue letters represent the genes of the MIF subfamily; Chr represents Chromosome, the number on the left indicates the length of the chromosome in megabase (Mb). (**B**) Ka/Ks values for duplicated *TaZF-HD* gene pairs. (**C**) Phylogeny of wheat and its genome ancestors. The phylogenetic tree was constructed using a Neighbor-joining (NJ) method. ZF-HDs from different species were distinguished with different color dots. (**D**) Homologous relationships between wheat and its three genome ancestors. The chromosomes of different species were shown in different color boxes. The numbers along each chromosome box indicate sequence lengths in Mb. Lines between two chromosomes represent the homologous relationships. (**E**) Synteny analysis of *ZF-HD* genes between wheat and four representative plant species. Including two dicotyledonous plants (*Arabidopsis* and tomato), and two monocotyledonous plants (rice and maize). Gray lines in the background indicate the collinear blocks within wheat and other plant genomes, while the red lines highlight the syntenic *ZF-HD* gene pairs.

Generally, gene duplications are considered to be one of the primary driving forces of species evolution [50]. Gene duplication includes segmental duplication and tandem duplication. Before analyzing the gene duplication events, we should determine the location of genes on the chromosome. Chromosome mapping revealed that the 37 *ZF-HD* genes were unevenly distributed across among the 18 chromosomes of wheat, there was no *ZF-HD* gene on 7 chromosomes (Fig 4A). While the 3A, 5A, 5B, and 5D chromosome had the highest number *ZF-HD* genes (four on each chromosome). Due to the incomplete sequencing of wheat genome, *TaZHD14-U* distributed on the unknown chromosome. There were 16 groups of gene duplication events, of which one group is a tandem duplication and the remaining groups are segmental duplication events (S5 Table). Moreover, there were some homologous genes, such as *TaZHD6-3A*, *TaZHD6-3B*, and *TaZHD6-3D*, derived from 3A, 3B, and 3D chromosomes of wheat, respectively. To better understand the evolutionary factors affecting *TaZF-HD* gene family, we calculated Ka/Ks (nonsynonymous substitution rates/synonymous substitution rates) value to infer the selection force during the evolution of genes [51]. In general, Ka/Ks > 1 represented positive selection of evolution acceleration; Ka/Ks = 1 represented neutral gene drift; and Ka/Ks < 1 represented purifying selection under functional constraints. The Ka/Ks values of the 13 *TaZF-HDs* homologous pairs were less than 1, and the values of other 3 pairs were 0 (Fig 4B and S6 Table). These results indicate that the gene family may have experienced powerful purification selection pressure during the process of wheat evolution to eliminate harmful mutations at the protein level, which may help to promote maintain the stability of the gene functions.

### Homologous gene pairs and synteny analysis of *TaZF-HDs*

In our research, 7, 22, 11 members of the *ZF-HD* family were identified in *T*. *urartu*, *T*. *dicoccoides*, and *Ae*. *Tauschii*, respectively. A comprehensive phylogenetic tree was established by NJ method (Fig 4C). Then a homologous analysis of wheat and three sub-genome donor species was performed, and the orthologs and paralogs were clustered. Orthologous refered that two genes in different species but derive from a common ancestor, paralogs described homologous genes within a single species and generated by gene duplication [52]. A total of 82 homologous genes were identified (Fig 4D), 65 homologous gene pairs were related to *T*. *aestivum*, of which 16 were paralogs gene pairs. Moreover, there were 9, 26, and 14 orthologs between *T*. *aestivum* and *T*. *urartu*, *T*. *dicoccoides*, and *Ae*. *Tauschii*.

In order to further analyze the evolution and homology relationship of the wheat *ZF-HD* family, the synteny analysis between wheat and other four species (*Arabidopsis*, rice, maize and tomato) were analyzed. The synteny analysis showed that there were multiple pairs of *ZF-HD* orthologous genes in wheat and other species except *Arabidopsis*. The number of orthologous genes were 23 (between wheat and rice), 36 (between wheat and maize), and 6 (between wheat and tomato) (Fig 4E). These genes may come from the same ancestral genes. Notably, there were more homologous genes between wheat and maize and rice (Wheat, maize, and rice are all monocotyledonous plants.), which is consistent with the evolutionary subordination of species.

### MiRNA targets and protein interaction network analysis of *TaZF-HD* genes

**Fig 5:**
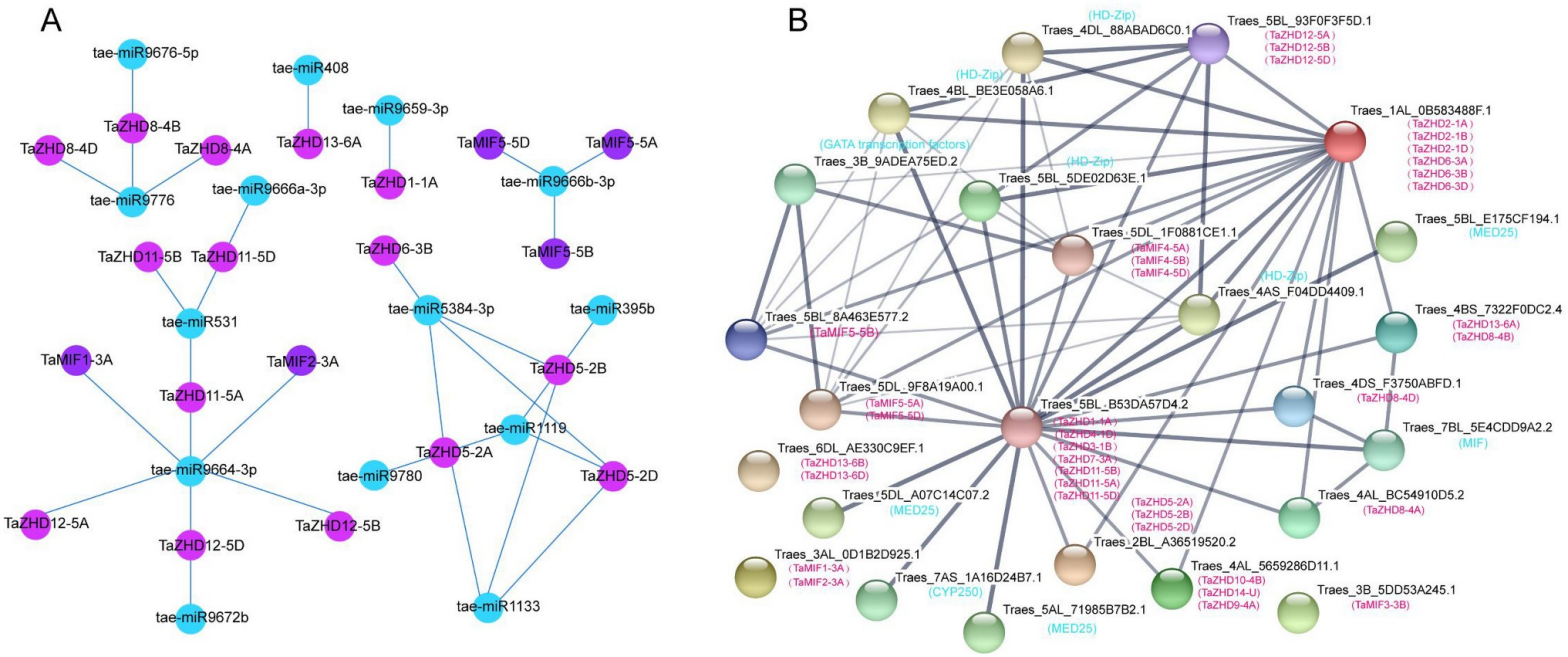
Protein interaction and miRNA targeting analysis. (**A**) The picture of the regulatory network relationships between the putative miRNAs and their targeted wheat ZF-HD genes. Different colored circles represent different types, blue represents miRNA, purple indicates predicted miRNA target genes. (**B**) Interaction networks of the wheat ZF-HD proteins.

MicroRNA (miRNA) is a class of non-coding single-stranded RNA molecules with a length of about 22 nucleotides encoded by endogenous genes. They have a variety of important regulatory effects in animal and plant cells, and can degrade mRNA of target gene or inhibit protein translation [53]. In the complex regulatory network of target genes and miRNAs, each miRNA can regulate multiple target genes, and a gene also could be precisely regulated by several miRNAs. The study of the regulatory relationship between miRNAs and genes will help us further understand the function of the target genes. Therefore, we systematically analyzed potential miRNAs that may target the wheat *ZF-HD* gene family and hope to provide information support for gene regulation mechanisms. We identified 14 miRNAs targeted to 20 *TaZF-HD* genes, and then used Cytoscape software to establish a relationship network (Fig 5A). By analyzing the regulatory network, we found that tae-miR9664-3p targets six TaZF-HD genes (*TaZHD12-5A*, *TaZHD12-5D*, *TaZHD12-5B*, *TaMIF1-3A*, *TaMIF2-3A*, *TaZHD11-5A*); tae-miR5384-3p targets four genes (*TaZHD5-2D*, *TaZHD5-2A*, *TaZHD5-2B*, *TaZHD6-3B*). Besides, tae-miR9776, tae-miR9666a-3p, tae-miR531, tae-miR1119 and tae-miR1133 have three targeted genes, respectively. Overall, by predicting the potential miRNAs of genes, understanding their important roles in plant development and stress resistance, has an important role in the study of gene function. According to the PPI prediction results (Fig 5B), we found 10 proteins that interact with the TaZF-HD proteins. Among these 10 proteins, 4 are homeobox leucine zipper (HD-Zip) protein, 3 are mediator complex 25 (MED25), and the remaining three proteins include a GATA-family transcription factors, a cytochrome P450 (CYP450) enzyme and a macrophage migration inhibitory factor (MIF). Besides, there are multiple interactions between TaZF-HD genes.

### Multiple conditional transcriptome analysis of *TaZF-HDs*

**Fig 6:**
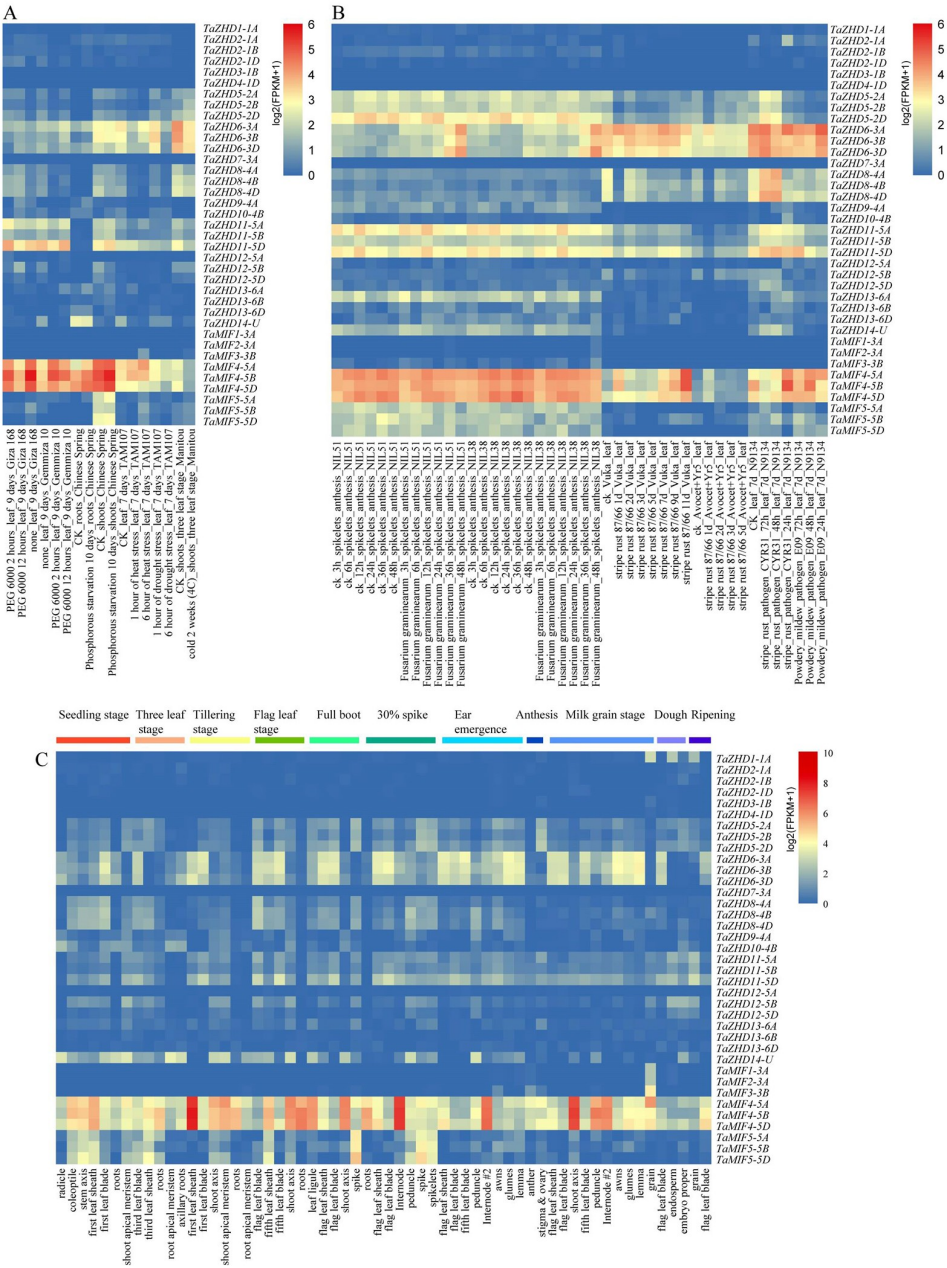
Multi-conditional transcriptome analysis of *TaZF-HDs* in different tissues and different biotic/abiotic stress environments. The R package ‘pheatmap’ was used to generate the heat maps. (**A**) Transcript level of *TaZF-HDs* responding to abiotic stresses; (**B**) Transcript level of *TaZF-HDs* responding to biotic stresses. (**C**) Transcript level of *TaZF-HDs* in wheat different tissues and development stages.

High-throughput technologies, such as RNA-Seq analysis, have been widely used for differential expression analysis [28]. In order to investigate the specific expression profiles of ZF-HD genes, the original RNA-seq data were downloaded. Then these data were sorted and expression heatmap of the TaZF-HD genes were generated using the R package ‘pheatmap’. As shown in Fig 6, three genes, *TaMIF4-5A*, *TaMIF4-5B*, and *TaMIF4-5D*, had the higher expression levels and were expressed in almost treatments and tissues in different wheat development stages, which indicated that these genes may play critical roles in wheat developmental stages or under various stresses. Besides, the expression levels of *TaZHD6-3A*, *TaZHD6-3B*, *TaZHD6-3D*, *TaZHD11-5D*, *TaZHD11-5A* and *TaZHD5-2D* were also relatively high in biotic stresses (*F*. *graminearum*, Powdery mildew, Stripe rust) and abiotic stresses (PEG6000, Phosphorous starvation, drought). In addition, most genes (such as *TaZHD1-1A*, *TaZHD2-1B*, *TaZHD9-4A* and *TaMIF1-3A*) had low expression levels under the various conditions and were expressed only in specific periods or under specific treatments, which indicated these genes may have potential functions.

### Real-time quantitative PCR and data analysis

**Fig 7:**
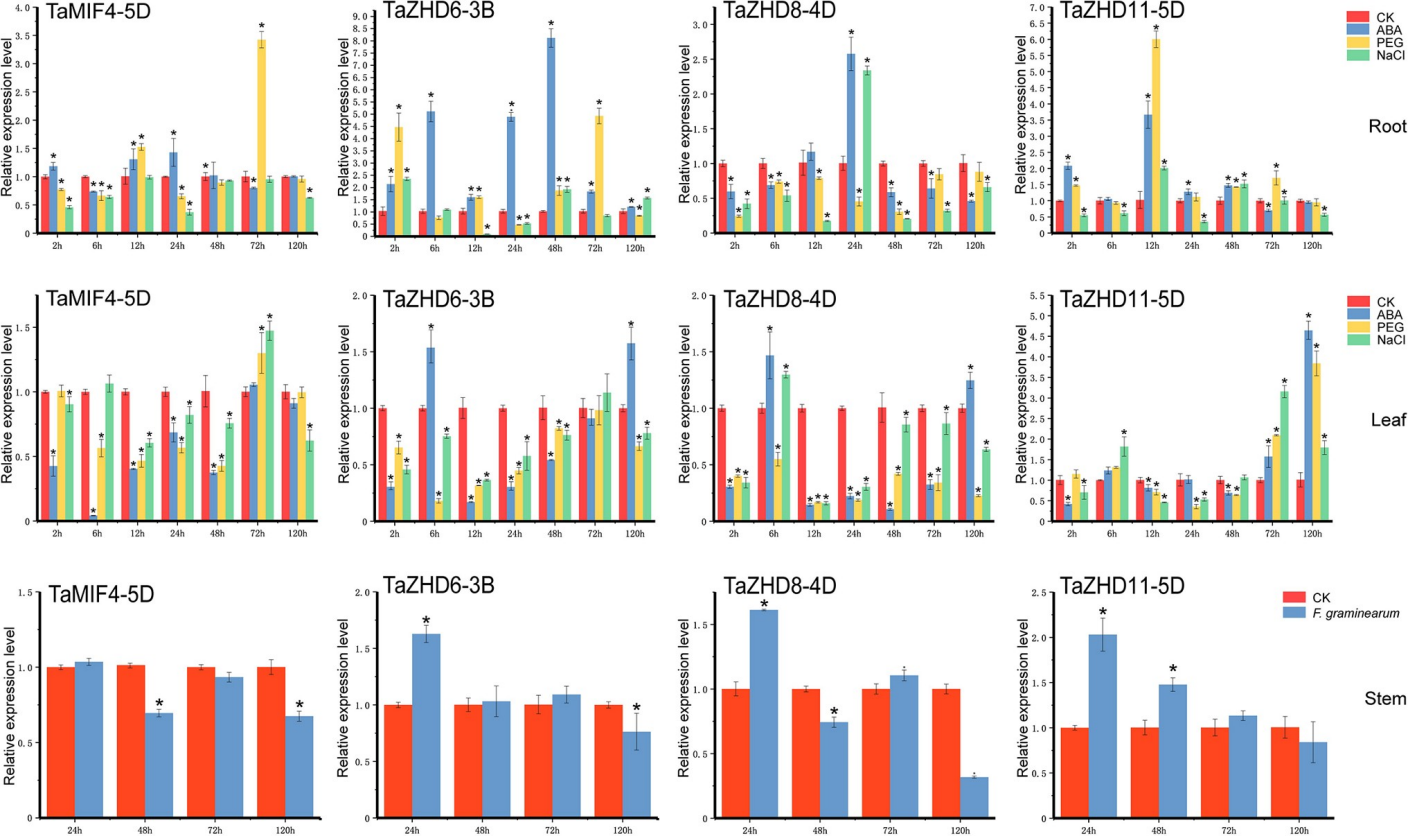
qRT-PCR analysis of *TaZF-HD* genes under biotic/abiotic stresses. The data were analyzed by three independent repeats, and standard deviations were shown with error bars. The expression level of *TaZF-HDs* genes was drawn using Origin software. * indicates significant differences that compared with control at *p* < 0.05.

The expression patterns of genes are often closely related to their functions. Our analysis indicated that most of the *ZF-HD* genes were expressed at low levels, but with tissue-specificity. In order to further reveal the expression level and potential functions of the *TaZF-HD* genes involved in biotic stresses *(F*. *graminearum*) and abiotic stresses (NaCl, PEG, and ABA), qRT-PCR was used to analyze expression patterns of four selected *TaZF-HD* genes (*TaMIF4-5D*, *TaZHD6-3B*, *TaZHD8-4D*, *TaZHD11-5D*). As shown in Fig 7, *TaMIF4-5D* was up-regulated at 12 h and 24 h after ABA treatment in root compared with control; The expression levels of *TaZHD6-3B* and *TaZHD11-5D* were higher than that of control, *TaZHD6-3B* had the highest expression at 48 h, while *TaZHD11-5D* had the highest expression at 12 h. Under ABA treatment in leaf, the results showed that the expression level of T*aMIF4-5D* was not significantly different with control at 72 h and 120 h, but lower than control at other points; the expression levels of *TaZHD6-3B* and *TaZHD8-4D* were up-regulated at 6 h and 120 h, while *TaZHD11-5D* was up-regulated at 72 h and 120 h. Under PEG-induced drought stress condition in root, the expression levels of *TaMIF4-5D* and *TaZHD11-5D* were up-regulated at 12 h and 72 h, and the expression level at other points were fluctuating. Compared with the control, the expression level of *TaZHD8-4D* was low at each stage. As for PEG stress in leaf, the expression of *TaMIF4-5D*, *TaZHD6-3B*, and *TaZHD8-4D* were lower than that of control, and expression of *TaZHD11-5D* was increased at 72 h and 120 h. In the case of NaCl stress in root, the expression of *TaMIF4-5D* was lower than that of control, *TaZHD8-4D* exhibited a significant improvement at 24 h, and the expression of *TaZHD11-5D* was up-regulated at 12 h and 48 h. Under NaCl stress in leaf, the expression of *TaMIF4-5D* was significantly up-regulated at 72 h, while the expression level of *TaZHD6-3B* was decreased than that of control throughout the treatment, *TaZHD11-5D* was up-regulated at 6 h, down-regulated at 12 h, and then but decreased at 120 hours. After inoculation with *F*. *graminearum*, the fluctuations of the expression levels of the four genes were light at different periods. The expression levels of *TaMIF4-5D* and *TaZHD8-4D* were down-regulated at 48 h and 120 h. The expression levels of *TaMIF4-5D* at other time points were not significantly different from the control, while the expression levels of *TaZHD8-4D* were up-regulated at 48 h and 72 h; the expression of *TaZHD6-3B* was increased than control at 24 h, while had no significant difference with control at 48 h and 72 h. The expression of *TaZHD11-5D* was increased at 24 h and 48 h compared with control, after that, the expression reduced to some extent. Considering, these results suggested that different *TaZF-HD* genes had various response to different stress treatments.

### Verification of subcellular localization and transcriptional autoactivation activity

**Fig 8:**
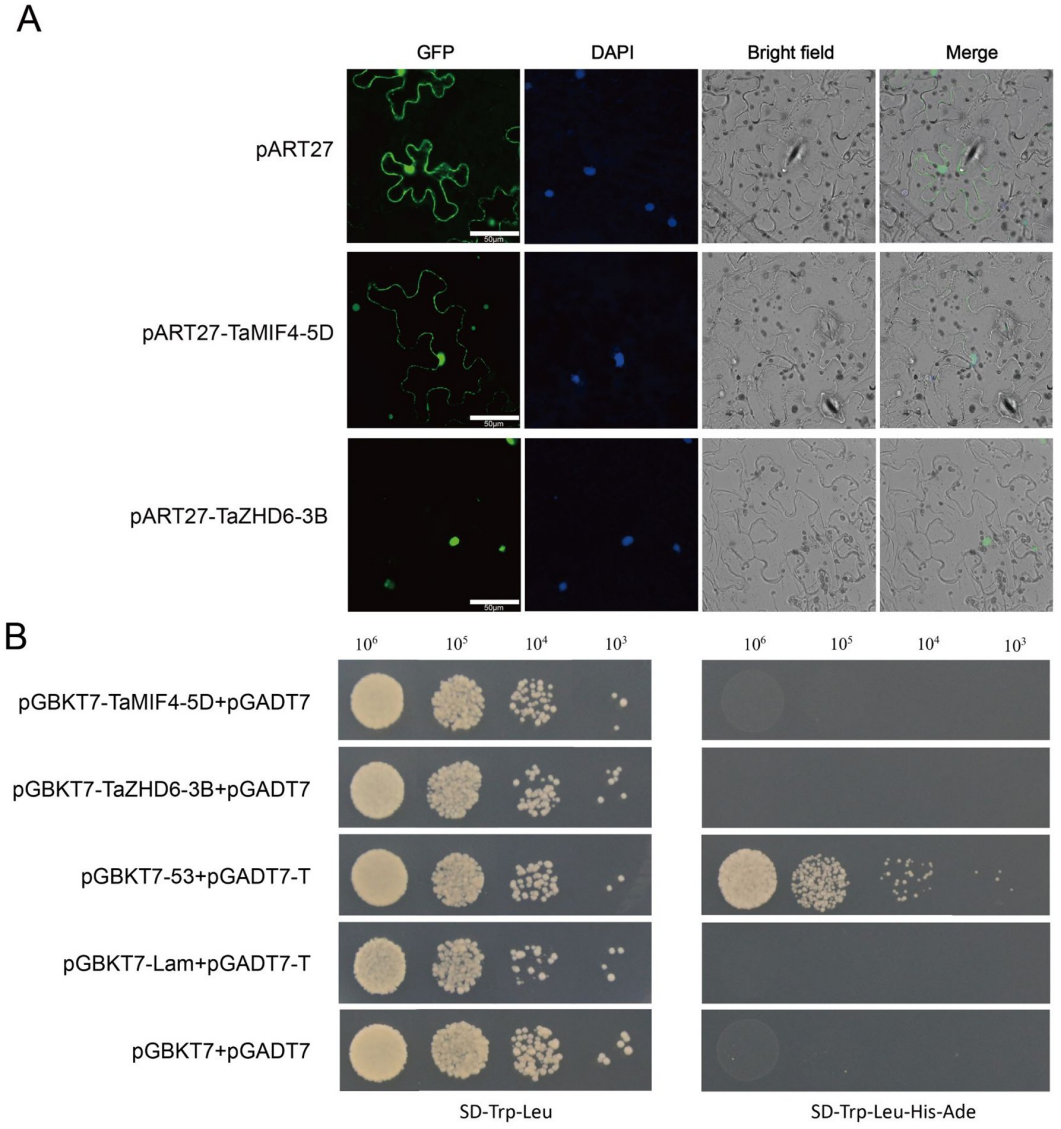
Subcellular localization and verification of transcriptional autoactivation activity of TaMIF4-5D and TaZHD6-3B. (**A**) Subcellular localization of TaMIF4-5D and TaZHD6-3B. The figure shows the fluorescence signal of GFP, the fluorescence signal after DAPI staining, the bright field and the fusion image, respectively. The fluorescent signal of GFP in the figure is used to mark the expression position of the fusion proteins. The fluorescent signal after DAPI staining is used to show the position of the cell nucleus. Scale bar = 50 μm. (**B**) Verification of TaMIF4-5D and TaZHD6-3B transcriptional autoactivation activity. The concentrations (cells/ml) of yeast transformants were adjusted to 10^6^, 10^5^,10^4^,10^3^, respectively.

Transcription factors is a class of DNA-binding proteins that can specifically interact with *cis*-elements in DNA or RNA, and can activate or inhibit the transcription and expression of certain genes. A typical transcription factor is composed of four functional regions: nuclear localization signal region (NLS), DNA binding region (BD), transcription control region and oligomerization site. In this study, the results of subcellular localizations of *TaMIF4-5D and TaZHD6-3B* are predicted to be in the nucleus. In order to further validate the location of *TaMIF4-5D* and *TaZHD6-3B*, we performed the transient expression in *N*. *benthiana*. Our study indicated that *TaZHD6-3B* was located in nucleus, while TaMIF4-5D was located in the cell membrane and nucleus (Fig 8A).

The transcriptional autoactivation activity result showed that yeast cells with fusion expression vectors pGBKT7-TaMIF4-5D and pGBKT7-TaZHD6-3B can grow on SD-Trp-Leu plates, but cannot grow on SD-Trp-Leu-His-Ade plates (Fig 8B), indicating that these two transcription factors have no transcriptional autoactivation activity.

## Discussion

As one of the most important transcription factor families, ZF-HD family has been reported in many plants. The ZF-HD family can be divided into ZHD subfamily and MIF subfamily, and they all have ZF domain, while the MIF subfamily lacks the HD domain. Unlike the ZHD gene, MIF is only found in seed plants. Previous study found that the ZHD gene appeared earlier than MIF in land plants, indicating that the original MIF gene may be derived from the ZHD gene [4]. Although previous studies have identified 17 in *Arabidopsis*, 15 in rice, 24 in maize, 20 in tartary buckwheat, 22 in tomato, and 31 in Chinese cabbage, there is a lack of research on the ZF-HD family in wheat. Based on the edible and economic value of wheat, it is necessary to study the function of ZF-HD family in wheat. In this study, 37 candidate *ZF-HD* genes were identified in wheat genome, including 28 members of *ZHD* subfamily, 9 members of *MIF* subfamily. Compared with *Arabidopsis*, rice, and maize, wheat has relatively more members in the *ZF-HD* family. In order to reveal the phylogenetic relationship of the ZF-HD family, a phylogenetic tree was constructed, and a total of 93 members from wheat, *Arabidopsis*, rice and maize were divided into six groups (Fig 1). In group 2 and group 5, there were genes from wheat, rice and maize, but no genes from *Arabidopsis*. It is speculated that this may be due to the separate evolution of the species after the differentiation of monocotyledon and dicotyledon.

Gene structure analysis is a key to reveal the function of genes. In present study, our results show that multiple *TaZF-HD* genes (30) do not contain intron, which is consistent with the results in *Arabidopsis*, rice, maize, and tartary buckwheat [18]. Studies have shown that genes with smaller or fewer introns may be more effectively expressed in stressful environments [54]. Therefore, these *TaZF-HD* genes may respond quickly under the external environment. For instance, *TaMIF4-5A*, *TaMIF4-5B*, and *TaMIF4-5D* had no introns, and they had efficient expression under various stresses (Figs 2B and 6). Besides, a total of 20 conserved motifs were identified in wheat ZF-HD family (Fig 2C). Among them, motif 1 present in all TaZF-HD members and correspond to the ZF domain, motif 2 and motif 3 correspond to the HD domain. In general, the members of ZF-HD family in the same group shared similar gene structure and motif composition, which indicates that they may play similar functions. Three genes (*TaMIF4-5A*, *TaMIF4-5B*, and *TaMIF4-5D*) were highly expressed in almost all selected wheat growth and development stage and stress treatments (Fig 6), which indicated that these genes may play important roles in growth and development. According to the analysis results of *cis*-acting elements, we found several mainly enriched elements of TaZF-HDs: TATA-box, G-box, CAAT-box, ABRE, TGA-element, GARE-motif and P-box, which were related to biotic/abiotic stress or plant growth and development. The multiple *cis*-elements of TaZF-HD gene promoters signify their regulatory effects on gene expression during different growth stages and under environmental stimuli.

In this study, a total of 16 duplication events with 19 *TaZF-HD* genes were found from wheat genome, of which 15 were segmental duplication. The Ka/Ks analysis of these gene pairs showed the selective power during the evolution of genes. In these duplication events, Ka/Ks value of 13 events was less than 1 (Fig 4B), which indicated that *ZF-HD* family may have experienced strong purification selection pressure during the evolution of wheat to eliminate harmful mutations at the protein level, which may help to promote the stability of the gene functions. The homologous analysis showed that 65 orthologous gene pairs were found between wheat and three sub-genome donor species. According to the syntenic analysis, there are 0, 23, 36, 6 orthologous gene pairs between wheat and *Arabidopsis*, rice, maize, tomato, respectively. These genes may come from same ancestral genes. Moreover, some *TaZF-HD* have orthologous gene pairs in tomato, maize, and rice, such as *TaZHD2-1D*, *TaZHD10-4B*, and *TaZHD6-3B*, indicating that these genes could already exist before speciation. Some *TaZF-HD* genes only found orthologous gene pairs in rice and maize, such as *TaZHD5-2A* and *TaZHD6-3B*, suggesting that these orthologous gene pairs appeared after the divergence of monocotyledonous and dicotyledonous plants.

As a small non-coding RNA, miRNA plays an important role in eukaryotes. Here, a total of 14 miRNAs were identified. Previous studies have shown that the expression of tae-miR9664 and its target genes play a key role in the resistance to wheat stripe rust [55], and we found that tae-miR9664 regulates six genes in TaZF-HDs. The prediction results of PPI found that there are interactions between members of the TaZF-HD family, and the TaZF-HDs also interacts with other proteins. It is speculated that the proteins of TaZF-HD family may regulate gene expression or participate in different physiological functions by forming homotypic or heterodimers.

Studies have shown that when plants are stimulated by unfavorable environments, ZF-HD gene expression usually responds to adversity [3]. In this study, qRT-PCR analysis was used to identify the expression patterns of four *TaZF-HD* genes under biotic and abiotic stresses. The results showed that the expression of the four genes (*TaMIF4-5D*, *TaZHD6-3B*, *TaZHD8-4D*, and *TaZHD11-5D*) were significantly down-regulated under *F*. *graminearum* inoculation, indicating that they played a critical role in responding to *F*. *graminearum* infection. In addition, these four genes were significantly up-regulated at different periods under ABA treatment, indicating that these genes can respond quickly to ABA stress. Three genes (*TaMIF4-5D*, *TaZHD6-3B*, and *TaZHD11-5D*) were up-regulated under PEG-simulated drought treatment, indicating that the three genes may have effects on responding to drought stress. *TaZHD8-4D* and *TaZHD11-5D* were up-regulated under NaCl treatment at a certain point, indicating that they may play roles in responding to salt stress (Fig 7).

Since transcription factors have nuclear localization signal regions (NLS), most transcription factors are located in the nucleus, but there are exceptions. For example, in *Arabidopsis*, the heat shock transcription factor *AtHsfA6a* was located in the cytoplasm [56]. We identified the TaZHD6-3B located in the nucleus, and the TaMIF4-5D located in the nucleus and cell membrane. The results showed that these two genes can be located in the nucleus, indicating that they may have functions as transcriptional factor. The transcriptional autoactivation experiment using yeast two-hybrid showed that both TaZHD6-3B and TaMIF4-5D have no autoactivation activity.

## Conclusion

In this experiment, 37 *TaZF-HD* genes were identified, which can be divided into ZHD subfamily and MIF subfamily according to the domains. The chromosome mapping result showed that except for *TaZHD14-U*, other genes are located on 18 wheat chromosomes. Ka/Ks analysis showed that the *TaZF-HD* family have experienced powerful purification selection during the evolution process. The qRT-PCR of four selected *TaZF-HD* genes showed that these genes almost all responded significantly to biotic and/or abiotic stresses, and different members showed different expression patterns. In addition, we have performed some basic molecular biology experiments to verify function of the *TaZF-HD* gene family members. The results of this study provide the original information and theoretical basis for the subsequent study of *TaZF-HDs*, and will lay a foundation to improve wheat quality traits in molecular breeding program.

## Supporting information

S1 Fig Conserved domain alignment of the ZF-HD family genes in wheat. (**A**) The ZF domain of the ZHD subfamily; (**B**) The HD domain of the ZHD subfamily; (**C**) The ZF domain of the MIF subfamily.
(TIF)

S1 Table ZF-HD gene information in 6 species. (XLSX)

S2 Table Multilevel consensus sequences in TaZF-HD genes identified by MEME. (XLSX)

S3 Table Numbers of cis-elements in upstream 1.5 kb regions of TaZF-HD genes. (XLSX)

S4 Table Location of TaZF-HD genes on chinese spring. (XLSX)

S5 Table Duplication of gene pairs among wheat and three donor species. (XLSX)

S6 Table Ka/Ks analysis for duplicated ZF-HD genes. (XLSX)

S7 Table qRT-PCR primers for TaZF-HD genes. (XLSX)

S8 Table Full-length primers for TaZF-HD genes. (XLSX)

S9 Table BLASTp search results. (XLSX)

## References

[1] Wingender E, Schoeps T, Haubrock M, Krull M, Dönitz J. TFClass: expanding the classification of human transcription factors to their mammalian orthologs. Nucleic Acids Research. 2018; 46 (D1): D343–D347. doi: 10.1093/nar/gkx987 29087517 PMC5753292

[2] Sharif R, Xie C, Wang J, Cao Z, Zhang HQ, Chen P, et al. Genome wide identification, characterization and expression analysis of HD-ZIP gene family in *Cucumis sativus* L. under biotic and various abiotic stresses. International Journal of Biological Macromolecules. 2020; 158: 502–520. doi: 10.1016/j.ijbiomac.2020.04.124 32376256

[3] Wang WL, Wu P, Li Y, Hou XL. Genome-wide analysis and expression patterns of ZF-HD transcription factors under different developmental tissues and abiotic stresses in Chinese cabbage. Molecular Genetics and Genomics. 2016; 291: 1451–1464. doi: 10.1007/s00438-015-1136-1 26546019

[4] Hu W, Claude WD, Ma H. Phylogenetic Analysis of the plant-specific zinc finger-homeobox and mini zinc finger gene families. Journal of Integrative Plant Bio. 2008; 50(8): 1031–1045. doi: 10.1111/j.1744-7909.2008.00681.x 18713354

[5] Mukherjee K, Brocchieri L, Bürglin TR. A comprehensive classification and evolutionary analysis of plant homeobox genes. Molecular Biology and Evolution. 2009; 26: 2775–2794. doi: 10.1093/molbev/msp201 19734295 PMC2775110

[6] Wolberger C. Homeodomain interactions. Current Opinion in Structural Biology. 1996; 6: 62–68. doi: 10.1016/s0959-440x(96)80096-0 8696974

[7] Bhattacharjee A, Ghangal R, Garg R, Jain M. Genome-wide analysis of homeobox gene family in legumes: Identification, gene duplication and expression profiling. PloS ONE. 2015 Mar; 10(3): e0119198. doi: 10.1371/journal.pone.0119198 25745864 PMC4352023

[8] Khatun K, Nath UK, Robin AHK, Park JI, Lee DJ, Kim MB, et al. Genome-wide analysis and expression profiling of zinc finger homeodomain (ZHD) family genes reveal likely roles in organ development and stress responses in tomato. BMC Genetics. 2017; 1: 695. doi: 10.1186/s12864-017-4082-y 28874115 PMC5585987

[9] Krishna SS, Majumdar I, Grishin NV. Structural classification of zinc fingers: survey and summary. Nucleic Acids Research. 2003; 31: 532–550. doi: 10.1093/nar/gkg161 12527760 PMC140525

[10] Windhovel A, Hein I, Dabrowa R, Stockhaus J. Characterization of a novel class of plant homeodomain proteins that bind to the C4 phosphoenolpyruvate carboxylase gene of *Flaveria trinervi*a. Plant Molecular Biology. 2001; 45: 201–214. doi: 10.1023/a:1006450005648 11289511

[11] Klug A, Schwabe JW. Protein motifs 5. Zinc fingers. FASEB Journal. 1995; 9(8): 597–604. 7768350

[12] Mackay JP, Crossley M. Zinc fingers are sticking together. Trends in Biochemical Sciences. 1998; 23: 1–4. doi: 10.1016/s0968-0004(97)01168-7 9478126

[13] Takatsuji H. Zinc-finger proteins: the classical zinc finger emerges in contemporary plant science. Plant Molecular Biology. 1999; 39(6): 1073–1078. doi: 10.1023/a:1006184519697 10380795

[14] Halbach T, Scheer N, Werr W. Transcriptional activation by the PHD finger is inhibited through an adjacent leucine zipper that binds 14-3-3 proteins. Nucleic acids research. 2000; 28(18): 3542–3550. doi: 10.1093/nar/28.18.3542 10982874 PMC110726

[15] Kosarev P, Mayer KF, Hardtke CS. Evaluation and classification of RING-finger domains encoded by the *Arabidopsis* genome. Genome Biology. 2002; 3 (4): 1–12. 10.1186/gb-2002-3-4-research0016 PMC115204 11983057

[16] Englbrecht CC, Schoof H, Böhm S. Conservation, diversification and expansion of C2H2 zinc finger proteins in the *Arabidopsis thaliana* genome. BMC Genomics. 2004; 5 (1): 1–17. doi: 10.1186/1471-2164-5-1 15236668 PMC481060

[17] Hu W, Ma H. Characterization of a novel putative zinc finger gene *MIF1*: involvement in multiple hormonal regulation of Arabidopsis development. The Plant Journal. 2006; 45(3): 399–422. doi: 10.1111/j.1365-313X.2005.02626.x 16412086

[18] Liu MY, Wang XX, Sun WJ, Ma ZT, Zheng TR, Huang L, et al. Genome-wide investigation of the ZF-HD gene family in Tartary buckwheat (*Fagopyrum tataricum*). BMC Plant Biology. 2019; 19(1): 248. doi: 10.1186/s12870-019-1834-7 31185913 PMC6558689

[19] Wang H, Yin XJ, Li XQ, Wang L, Zheng Y, Xu XZ, et al. Genome-Wide identification, evolution and expression analysis of the grape (*Vitis vinifera* L.) zinc finger-homeodomain gene family. International Journal of Molecular Sciences. 2014; 15(4): 5730–5748. doi: 10.3390/ijms15045730 24705465 PMC4013592

[20] Abdullah M, Cheng X, Cao Y, Su X, Manzoor MA, Gao J, et al. Zinc finger-homeodomain transcriptional factors (ZHDs) in upland cotton (*Gossypium hirsutum*): Genome-wide identification and expression analysis in fiber development. Frontiers in Genetics. 2018; 9: 357. doi: 10.3389/fgene.2018.00357 30356782 PMC6189526

[21] Li CY. Identification and characterization of genes related to salt tolerance and drought resistance of the ZF-HD transcription factor family in maize. Southwest University, Chongqing, China. 2018. CNKI:CDMD:2.1018.860139.

[22] Figueiredo D, Barros P, Cordeiro A, Serra T, Lourenço T, Chander S, et al. Seven zinc-finger transcription factors are novel regulators of the stress responsive gene OsDREB1B. Journal of experimental botany. 2012; 63(10): 3643–3656. doi: 10.1093/jxb/ers035 22412187

[23] Tran LS, Nakashima K, Sakuma Y, Osakabe Y, Qin F, Simpson SD, et al. Co-expression of the stress-inducible zinc finger homeodomain *ZFHD1* and *NAC* transcription factors enhances expression of the *ERD1* gene in *Arabidopsis*. Plant Journal for Cell & Molecular Biology. 2007; 49(1): 46–63. doi: 10.1111/j.1365-313X.2006.02932.x 17233795

[24] Shalmani A, Muhammad I, Sharif R, Zhao C, Ullah U, Zhang D, et al. Zinc finger-homeodomain genes: evolution, functional differentiation, and expression profiling under flowering-related treatments and abiotic stresses in plants. Evolutionary Bioinformatics Online. 2019; 15. doi: 10.1177/1176934319867930 31523124 PMC6728664

[25] Queenie KG, Vivian FI. The Arabidopsis zinc finger-homeodomain genes encode proteins with unique biochemical properties that are coordinately expressed during floral development. Plant Physiology. 2006; 140 (3): 1095–1108. doi: 10.1104/pp.105.070565 16428600 PMC1400567

[26] Park HC, Kim ML, Lee SM, Bahk JD, Yun DJ, Lim CO, et al. Pathogen-induced binding of the soybean zinc finger homeodomain proteins GmZF-HD1 and GmZF-HD2 to two repeats of ATTA homeodomain binding site in the calmodulin isoform 4 (*GmCaM4*) promoter. Nucleic Acids Research. 2007; 35(11): 3612–3623. doi: 10.1093/nar/gkm273 17485478 PMC1920248

[27] Fang ZW, Sun C, Lu T, Xu Z, Huang WD, Ma DF, et al. Molecular mapping of stripe rust resistance gene YrH922 in a derivative of wheat (*Triticum aestivum*)–*Psathyrostachys huashanica*. Crop & Pasture Science. 2019; 70: 939–945. doi: 10.1071/CP19317

[28] Yin JL, Fang ZW, Sun C, Zhang P, Zhang X, Lu C, et al. Rapid identification of a stripe rust resistant gene in a space-induced wheat mutant using specific locus amplified fragment (SLAF) sequencing. Scientific Reports. 2018; 8(1): 3086. doi: 10.1038/s41598-018-21489-5 29449594 PMC5814476

[29] Chen SH, Jiang WQ, Yin JL, Wang SP, Fang ZW, Ma DF, et al. Genome-wide mining of wheat B-BOX zinc finger (BBX) gene family provides new insights into light stress responses. Crop and Pasture Science. 2021; 72: 17–37. doi: 10.1071/CP20342

[30] Artimo P, Jonnalagedda M, Arnold K, Baratin D, Csardi G, De Castro E, et al. ExPASy: SIB bioinformatics resource portal. Nucleic Acids Research. 2012; 40: 597–603. doi: 10.1093/nar/gks400 22661580 PMC3394269

[31] Chou KC, Shen HB. Plant-mPLoc: A Top-Down strategy to augment the power for predicting plant protein subcellular localization. PLOS ONE. 2010, 5 (6): e11335. doi: 10.1371/journal.pone.0011335 20596258 PMC2893129

[32] Bailey TL, Johnson JR, Grant CE, Noble WS. The MEME Suite. Nucleic Acids Research. 2015; 43: 39–49. doi: 10.1093/nar/gkv416 25953851 PMC4489269

[33] Chen CJ, Xia R, Chen H, He YH. TBtools, a Toolkit for biologists integrating various HTS-data handling tools with a user-friendly interface. BioRxiv. 2018.

[34] Hu LP, Zhang F, Song SH, Tang XW, Xu H, Liu GM, et al. Genome-wide identification, characterization, and expression analysis of the *SWEET* gene family in cucumber. Journal of Integrative Agriculture. 2017; 16: 1486–1501.

[35] Lescot M, Déhais P, Thijs G, Marchal K, Moreau Y, Vande PY, et al. PlantCARE, a database of plant cis-acting regulatory elements and a portal to tools for in silico analysis of promoter sequences. Nucleic Acids Research. 2002; 30(1): 325–327. doi: 10.1093/nar/30.1.325 11752327 PMC99092

[36] Fang ZW, Jiang WQ, He YQ, Ma DF, Liu YK, Wang SP, et al. Genome-Wide identification, structure characterization, and expression profiling of Dof transcription factor gene family in wheat (*Triticum aestivum* L.). Agronomy. 2020; 10(2): 294. doi: 10.3390/agronomy10020294

[37] Rogers RL, Cridland JM, Shao L, H TT, Andolfatto P, Thornton KR. Landscape of standing variation for tandem duplications in *Drosophila yakuba* and *Drosophila simulans*. Molecular Biology and Evolution. 2014; 31: 1750–1766. doi: 10.1093/molbev/msu124 24710518 PMC4069613

[38] Rogers RL, Ling S, Thornton KR, Begun DJ. Tandem duplications lead to novel expression patterns through exon shuffling in *drosophila yakuba*. PLoS Genetics. 2017; 13, e1006795. doi: 10.1371/journal.pgen.1006795 28531189 PMC5460883

[39] Zhou XY, Zhu XG, Shao WN, Song JH, Jiang WQ, He YQ, et al. Genome-wide mining of wheat *DUF966* gene family provides new insights into salt stress responses. Frontiers in Plant Science. 2020; 11. doi: 10.3389/fpls.2020.569838 32983219 PMC7483657

[40] Wang YP, Tang HB, DeBarry JD, Xu T, Li JP, Wang XY, et al. Mcscanx: a toolkit for detection and evolutionary analysis of gene synteny and collinearity. Nucleic Acids Research 2012;40 (7): e49. doi: 10.1093/nar/gkr1293 22217600 PMC3326336

[41] Dai XB, Zhao XC. psRNATarget: a plant small RNA target analysis server. Nucleic Acids Research. 2011; 39(2): W155–W159. doi: 10.1093/nar/gkr319 21622958 PMC3125753

[42] Otasek D, Morris JH, Bouças J, Pico AR, Demchak B. Cytoscape Automation: empowering workflow-based network analysis. Genome Biology. 2019; 20(1). doi: 10.1186/s13059-019-1758-4 31477170 PMC6717989

[43] Zhao J, Wang Y, Li X, Gai Z. Genome-wide identification and characterization of Toll-like receptors (TLRs) in housefly (*Musca domestica*) and their roles in the insecticide resistance. International Journal of Biological Macromolecules. 2020; 150: 141–151. doi: 10.1016/j.ijbiomac.2020.02.061 32045613

[44] He YQ, H WD, Yang L, L YT, Lu C, Zhu YX, et al. Genome-wide analysis of ethylene-insensitive3 (EIN3/EIL) in *Triticum aestivum*. Crop Science. 2020; 60(4). 60. doi: 10.1002/csc2.20115

[45] Trapnell C, Roberts A, Goff L, Pertea G, Kim D, Kelley DR, et al. Differential gene and transcript expression analysis of RNA-seq experiments with TopHat and Cufflinks. Nature Protocols. 2012; 7(3): 562–578. doi: 10.1038/nprot.2012.016 22383036 PMC3334321

[46] Ma DF, Yin JL, Chao KX, Jing JX, Li Q, Wang BT. Molecular mapping of stripe rust resistance gene YrHu derived from *Psathyrostachys huashanica*. Molecular Breeding. 2016; 36(6): 36–64. doi: 10.1007/s11032-016-0487-6

[47] Zhu YX, Xu XB, Hu YH, Han WH, Yin JL, Li HL, et al. Silicon improves salt tolerance by increasing root water uptake in *Cucumis sativus* L. Plant Cell Reports, 2015; 34(9): 1629–1646. doi: 10.1007/s00299-015-1814-9 26021845

[48] Livak KJ, Schmittgen TD. Analysis of relative gene expression data using real-time quantitative PCR and the 2(-Delta Delta C(T)) method. Methods, 2001; 25(4), 402–408. doi: 10.1006/meth.2001.1262 11846609

[49] Wittkopp PJ, Kalay G. Cis-regulatory elements: molecular mechanisms and evolutionary processes underlying divergence. Nature Reviews Genetics. 2012; 13(1): 59–69. doi: 10.1038/nrg3095 22143240

[50] Cannon SB, Mitra A, Baumgarten A, Young ND, May G. The roles of segmental and tandem gene duplication in the evolution of large gene families in *Arabidopsis thaliana*. BMC Plant Biology. 2004; 4(1): 796–815. doi: 10.1186/1471-2229-4-10 15171794 PMC446195

[51] Peterson GI, Masel J. Quantitative prediction of molecular clock and Ka/Ks at short timescales. Molecular Biology and Evolution. 2009; 26(11): 2595–2603. doi: 10.1093/molbev/msp175 19661199 PMC2912466

[52] Sonnhammer EL, Koonin EV. Orthology, paralogy and proposed classification for paralog subtypes. Trends in Genetics. 2003; 18(12): 619–620. doi: 10.1016/s0168-9525(02)02793-2 12446146

[53] Zhu YX, Jia JH, Yang L, Xia YC, Zhang HL, Jia JB, et al. Identification of cucumber circular RNAs responsive to salt stress. BMC Plant Biology. 2019c; 19(1): 164. doi: 10.1186/s12870-019-1712-3 31029105 PMC6486992

[54] Heyn P, Kalinka A, Tomancak P, Neugebauer K. Introns and gene expression: cellular constraints, transcriptional, regulation and evolutionary consequences. BioEssays. 2015; 37(5): 148–154. doi: 10.1002/bies.201400138 25400101 PMC4654234

[55] Ramachandran SR, Mueth NA, Zheng P, Hulbert SH. Analysis of miRNAs in two wheat cultivars infected with *puccinia striiformis* f. sp. *tritici*. Front Plant Science. 2019; 10: 1574. doi: 10.3389/fpls.2019.01574 31998329 PMC6965360

[56] Yao X, Gong ZH, Lu MH, Ma C, Li DW, Zhao J, et al. Cloning and cell location analysis of *Arabidopsis* heat shock transcription factor *AtHsfA6a*. Journal of Northwest A&F University: Natural Science Edition. 2008; 36(2): 94–98.

