# Supplementary figures and images for: Genome-wide identification of ZF-HD gene family in Triticum aestivum: Molecular evolution mechanism and function analysis
Source: PLoS One. 2021 Sep 24;16(9):e0256579. doi: 10.1371/journal.pone.0256579 (PMC8462724; doi:10.1371/journal.pone.0256579)

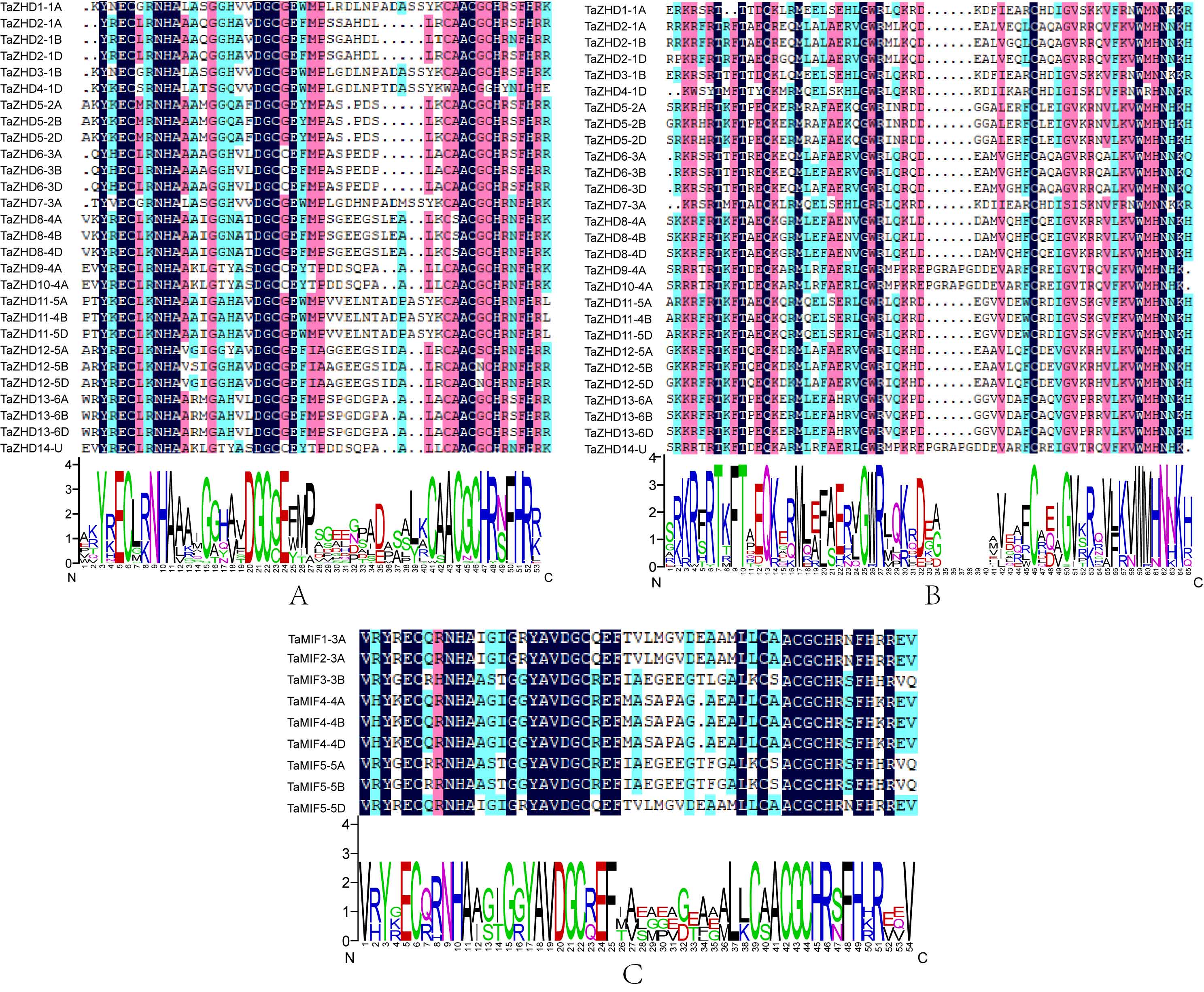

Supplement: S1 Fig — (A) The ZF domain of the ZHD subfamily; (B) The HD domain of the ZHD subfamily; (C) The ZF domain of the MIF subfamily. (TIF) [file pone.0256579.s001.tif]
